# Histogram analysis parameters of dynamic contrast-enhanced magnetic resonance imaging can predict histopathological findings including proliferation potential, cellularity, and nucleic areas in head and neck squamous cell carcinoma

**DOI:** 10.18632/oncotarget.24920

**Published:** 2018-04-20

**Authors:** Alexey Surov, Hans Jonas Meyer, Leonard Leifels, Anne-Kathrin Höhn, Cindy Richter, Karsten Winter

**Affiliations:** ^1^ Department of Diagnostic and Interventional Radiology, University Hospital of Leipzig, 04103 Leipzig, Germany; ^2^ Department of Pathology University Hospital of Leipzig, 04103 Leipzig, Germany; ^3^ Institute of Anatomy, University Hospital of Leipzig, 04103 Leipzig, Germany

**Keywords:** histogram analysis parameters, DCE MRI, proliferation index, cell count, head and neck squamous cell carcinoma

## Abstract

Our purpose was to analyze possible associations between histogram analysis parameters of dynamic contrast-enhanced magnetic resonance imaging DCE MRI and histopathological findings like proliferation index, cell count and nucleic areas in head and neck squamous cell carcinoma (HNSCC).

30 patients (mean age 57.0 years) with primary HNSCC were included in the study. In every case, histogram analysis parameters of K_trans_, V_e_, and K_ep_ were estimated using a mathlab based software. Tumor proliferation index, cell count, and nucleic areas were estimated on Ki 67 antigen stained specimens. Spearman's non-parametric rank sum correlation coefficients were calculated between DCE and different histopathological parameters.

KI 67 correlated with K_trans_ min (*p* = −0.386, *P* = 0.043) and s K_trans_ skewness (*p* = 0.382, *P* = 0.045), V_e_ min (*p* = −0.473, *P* = 0.011), Ve entropy (*p* = 0.424, *P* = 0.025), and K_ep_ entropy (*p* = 0.464, *P* = 0.013). Cell count correlated with K_trans_ kurtosis (*p* = 0.40, *P* = 0.034), V_e_ entropy (*p* = 0.475, *P* = 0.011). Total nucleic area correlated with V_e_ max (*p* = 0.386, *P* = 0.042) and V_e_ entropy (*p* = 0.411, *P* = 0.030).

In G1/2 tumors, only K_trans_ entropy correlated well with total (*P* =0.78, *P* =0.013) and average nucleic areas (*p* = 0.655, *P* = 0.006). In G3 tumors, KI 67 correlated with Ve min (*p* = −0.552, *P* = 0.022) and V_e_ entropy (*p* = 0.524, *P* = 0.031). Ve max correlated with total nucleic area (*p* = 0.483, *P* = 0.049). Kep max correlated with total area (*p* = −0.51, *P* = 0.037), and K_ep_ entropy with KI 67 (*p* = 0.567, *P* = 0.018).

We concluded that histogram-based parameters skewness, kurtosis and entropy of K_trans_, V_e_, and K_ep_ can be used as markers for proliferation activity, cellularity and nucleic content in HNSCC. Tumor grading influences significantly associations between perfusion and histopathological parameters.

## INTRODUCTION

Dynamic contrast-enhanced magnetic resonance imaging (DCE MRI) is a modality to characterize perfusion and vascularization of tissues [1–3]. According to the literature, parameters of DCE MRI can differentiate between malignant and benign lesions in several organs [1, 3]. DCE MRI can also distinguish low and high grade tumors [4–6]. Furthermore, some reports identified significant associations between DCE MRI findings and histopathology in several malignancies [7–9]. So Li *et al.* showed that the perfusion parameters significantly correlated with microvessel density in breast cancer [7]. Additionally, Jain *et al.* found that DCE MRI was associated with proliferation index KI 67 in glioma [8]. In HNSCC, it has been shown that DCE MRI parameters reflected well microvessel density [9].

Currently, a new approach of imaging analysis, namely histogram analysis of different radiological parameters is in trend. So some reports indicated that histogram analysis parameters of apparent diffusion coefficient (ADC) can better reflect several morphological features in different malignancies in comparison to established ADC parameters [10–12]. For instance, Liu *et al.* showed that ADC histogram parameters can well differentiate T and N stages in gastic cancer [13]. Also histogram parameters of DCE MRI have been reported to have a great diagnostic potential. According to Lee *et al.*, histogram parameters of MR perfusion can distinguish between oligodendroglioma and astrocytic tumors [14]. Furthermore, histogram DCE MRI parameters can be used to differentiate between lymphoma and squamous cell carcinoma of the oropharynx [15].

Presumably, histogram based DCE MRI parameters are more sensitive to reflect histopathololgical features than mean values used in clinical practice.

The purpose of this study was to analyze possible associations between histogram analysis parameters of DCE MRI and histopathological findings like proliferation index, cell count and nucleic areas in HNSCC.

## RESULTS

Correlation analysis identified several statistically significant correlations and correlation trends between the investigated parameters. KI 67 correlated with K_trans_ min (*P* = −0.386, *P* = 0.043) and K_trans_ skewness (*P* = 0.382, *P* = 0.045) (Table 1). Furthermore, K_trans_ kurtosis correlated with cell count (*P* = 0.40, *P* = 0.034). V_e_ min correlated with KI 67 (*P* = −0.473, *P* = 0.011) and V_e_ max with total nucleic area (*P* = 0.386, *P* = 0.042). V_e_ entropy showed significant correlations with KI 67 (*P* = 0.424, *P* = 0.025), cell count (*P* = 0.475, *P* = 0.011), and total nucleic area (*P* = 0.411, *P* = 0.030). Finally, K_ep_ entropy correlated with KI 67 (*P* = 0.464, *P* = 0.013).

**Table 1:** Correlations between DCE MRI and histopathological parameters

Parameters	Ki67	Cell count	Total nucleic area	Average nucleic area
**K_trans_ min**	*p* = −0.386 *P* = 0.043			*p* = −0.347 *P* = 0.07
**K_trans_ P10**	*p* = −0.334 *P* = 0.083			
**Median**	*p* = −0.321 *P* = 0.096			
**K_trans_ kurtosis**	*p* = 0.358 *P* = 0.06	*p* = 0.40 *P* = 0.034		
**K_trans_ skewness**	*p* = 0.382 *P* = 0.045	*p* = 0.371 *P* = 0.052		
**Ve min**	*p* = −0.473 *P* = 0.011			
**V_e_ max**			*p* = 0.386 *P* = 0.042	
**V_e_ P10**	*p* = −0.365 *P* = 0.056			
**V_e_ P25**	*p* = −0.359 *P* = 0.061			
**V_e_ median**	*p* = −0.328 *P* = 0.088			
**V_e_ mode**	*p* = −0.338 *P* = 0.079			
**V_e_ entropy**	*p* = 0.424 *P* = 0.025	*p* = 0.475 *P* = 0.011	*p* = 0.411 *P* = 0.030	
**K_ep_ entropy**	*p* = 0.464 *P* = 0.013	*p* = 0.321 *P* = 0.096		

Only statistically significant correlations and statistical trends are shown.

On the next step, separate correlation analyses in the low and high grade HNSCC were performed. In G1/2 tumors, only K_trans_ entropy correlated well with total (*P* = 0.78, *P* = 0.013) and average nucleic areas (*P* = 0.655, *P* = 0.006) (Table 2). There were no other statistically significant correlations or correlation trends between the parameters.

**Table 2:** Correlations between DCE MRI and histopathological parameters in G1/2 tumors

Parameters	Total nucleic area	Average nucleic area
**K_trans_ kurtosis**		
**K_trans_ entropy**	*p* = 0.78 *P* = 0.013	*p* = 0.655 *P* = 0.006

Only statistically significant correlations and statistical trends are shown.

In G3 tumors, KI 67 correlated with V_e_ min (*P* = −0.552, *P* = 0.022) and V_e_ entropy (*P* = 0.524, *P* = 0.031) (Table 3). Furthermore, V_e_ max correlated with total nucleic area (*P* = 0.483, *P* = 0.049). In addition, K_ep_ max correlated with total area (*P* = −0.51, *P* = 0.037), and K_ep_ entropy with KI 67 (*P* = 0.567, *P* = 0.018).

**Table 3:** Associations between DCE MRI and histopathological parameters in G3 tumors

Parameters	Ki67	Total nucleic area	Average nucleic area
**K_trans_ mean**	*p* = −0.415 *P* = 0.097		
**K_trans_ P10**	*p* = −0.439 *P* = 0.078		
**V_e_ min**	*p* = −0.552 *P* = 0.022		
**V_e_ max**	*p* = 0.417 *P* = 0.096	*p* = 0.483 *P* = 0.049	
**V_e_ P10**	*p* = −0.443 *P* = 0.075		
**V_e_ entropy**	*p* = 0.524 *P* = 0.031	*p* = 0.441 *P* = 0.076	
**K_ep_ max**		*p* = −0.51 *P* = 0.037	*p* = −0.448 *P* = 0.072
**K_ep_ entropy**	*p* = 0.567 *P* = 0.018		

Only statistically significant correlations and statistical trends are shown

## DISCUSSION

The present study identified associations between different DCE MRI parameters derived from histogram analysis and histopathological findings in HNSCC.

As mentioned above, previously, numerous studies analyzed DCE MRI findings in HNSCC [16–21]. It has been shown that perfusion parameters predicted tumor behavior and, therefore, can be used as biomarker [16–21]. So far volume of V_e_ is one of the independent prognostic factors for neck control in HNSCC treated with chemoradiation [16]. Furthermore, Kim *et al.* investigated lymph node metastases in HNSCC and found that low pretreatment K_trans_ was associated with a poor response to concurrent chemoradiation therapy [18]. Finally, Chawla *et al.* showed that lower pretreatment K_trans_ correlated with shorter disease-free survival [19].

The reported results suggested that DCE MRI parameters should be associated with relevant histopathological features in HNSCC. Previously, only two studies analyzed possible relationships between DCE MRI and histopathology in patients HNSCC [9, 22]. So Jansen *et al.* identified statistically significant correlations between KI 67 and K_trans_, V_e_, as well between K_ep_ and vascular endothelial growth factor (VEGF) in neck nodal metastases of HNSCC [22]. However, other authors did not find statistically significant correlations between DCE MRI parameters and KI 67 or tumor cellularity in HNSCC [9]. Presumably, these controversial results may be related to the fact that the studies investigated different parameters: in one study [22] standard deviation and in another [9] mean values of DCE parameters were analyzed. There is also another problem, namely the previous analyses investigated small number of patients, namely 12 [22] and 16 [9]. This fact also relativizes the reported results.

We hypothesize that DCE MRI should have more significant correlations with histopathology than reported previously. Especially V_e_ and K_ep_ should be associated with cellularity. This suggestion seems to be logical. In fact, V_e_ represents volume of the extravascular extracellular space [23, 24] and, therefore, may be associated with cell count. Furthermore, the assumed correlation between V_e_ and cellularity should be inversely, namely more cells-less extracellular space and vice versa. In fact, in an experimental study with glioma model, a strong inverse correlation (*r* = −0.75) between V_e_ and cellularity could be identified [25].

Similarly, K_ep_ reflects diffusion of contrast medium from the extravascular extracellular space back to the plasma [23, 24], and may also have relationships with tumor cellularity. Previous studies indicated correlations between K_trans_ and microvessel density in several malignancies [7, 9]. Also some authors suggested that K_trans_ correlated with proliferation activity (KI 67 expression) in hepatocellular carcinoma [26]. However, this did not apply for all lesions [8, 9]. On the other hand, K_trans_ reflects diffusion of contrast medium from the plasma through the vessel wall into the interstitial space [23, 24], and, therefore, may also have significant correlations with tumor cell count.

The present study confirmed these assumptions. Here, interestingly phenomena were identified. Proliferation activity slightly correlated with the minimum of K_trans_ and V_e_ and tended to correlate with several percentiles of both parameters. Furthermore, stronger correlations were observed between histogram-based parameters skewness, kurtosis and entropy and different histopathological findings. So kurtosis of K_trans_ correlated statistically significant with cell count and tended to correlate with KI 67, and skewness of K_trans_ correlated with KI 67. Entropy of V_e_ correlated with KI 67 expression, cell count, and total nucleic area. Finally, entropy of K_ep_ correlated with KI 67. In contrast to the previous reports, neither standard deviation nor mean values of the investigated perfusion parameters showed significant correlations with histopathological findings. These findings indicated the following: firstly, routinely used perfusion parameters (mean, median or standard deviation) are non sensitive to reflect relationships with cellularity and proliferation activity in HNSCC. Secondly, histogram-based parameters are better associated with proliferation activity, cellularity and nucleic areas in HNSCC. This finding is very important for clinical practice and implicates use of histogram-based DCE MRI parameters to predict histopathological features in HNSCC.

Furthermore, our study identified also another phenomenon, namely associations between perfusion parameters and histopathology is different in different tumor grades. Significantly more correlations and trends are seen in G3 tumors than in low grade lesions. The exact cause of this finding is unclear. Presumably, high grade carcinomas have other architecture, including relationships between tumor cells, especially cell size or volume and extracellular space, as well nucleic-cytoplasmic ratio. Also other features like stroma and microvessel density etc. may also play a role. These factors results in different associations between histopathology and perfusion parameters derived from DCE MRI. Clearly, further works with more cases are needed to confirm this hypothesis. Also search for relevant associations between histogram analysis DCE MRI parameters and other histopathological features like invasiveness should be performed in HNSCC.

In conclusion, histogram analysis DCE MRI parameters are sensitiver than mean DCE MRI values to reflect associations with histopathology in HNSCC. Especially, histogram-based parameters skewness, kurtosis and entropy of K_trans_, V_e_, and K_ep_ can be used as surrogate markers for proliferation activity, cellularity and nucleic content in HNSCC. Tumor grading influences significantly associations between perfusion parameters and histopathologic findings.

## MATERIALS AND METHODS

This study was IRB-approved and all patients gave their written informed consent.

### Patients

Overall, 30 patients (8 women and 22 men, mean age 57.0 ± 10.6 years, range, 33-77 years) with histological proven primary HNSCC were included in the study. The diagnosed tumors were localized in the oropharynx (46.7%), tongue (23.3%), hypopharynx (10%), larynx (16.7%), and nasopharynx (3.3%). Most frequently, the identified lesions were staged as T3 (33.3%) or T4 tumors (40%) with additional nodal (90%) metastases (Table 4). G1/2 tumors were diagnosed in 36.7% and G3 lesions in 63.3%.

**Table 4:** Localization and stage of the identified tumors

Diagnosis	*n* (%)
Carcinoma of nasopharynx	1 (3.3)
Carcinoma of oropharynx	14 (46.6)
Carcinoma of hypopharynx	3 (10)
Carcinoma of larynx	5 (16.7)
Carcinoma of tongue	7 (23.3)
**Tumor stage**	***n* (%)**
T stage	
T1	1 (3.3)
T2	7 (23.3)
T3	10 (33.3)
T4	12 (40)
**N stage**	***n* (%)**
N0	3 (10)
N1	5 (16.7)
N2	19 (63.3)
N3	3 (10)
**M stage**	***n* (%)**
M0	28 (93.3)
M1	2 (6.7)
**Tumor grading**	***n* (%)**
G1	1 (3.3)
G2	10 (33.3)
G3	19 (63.3)

### DCE MRI

**Figure 1:**
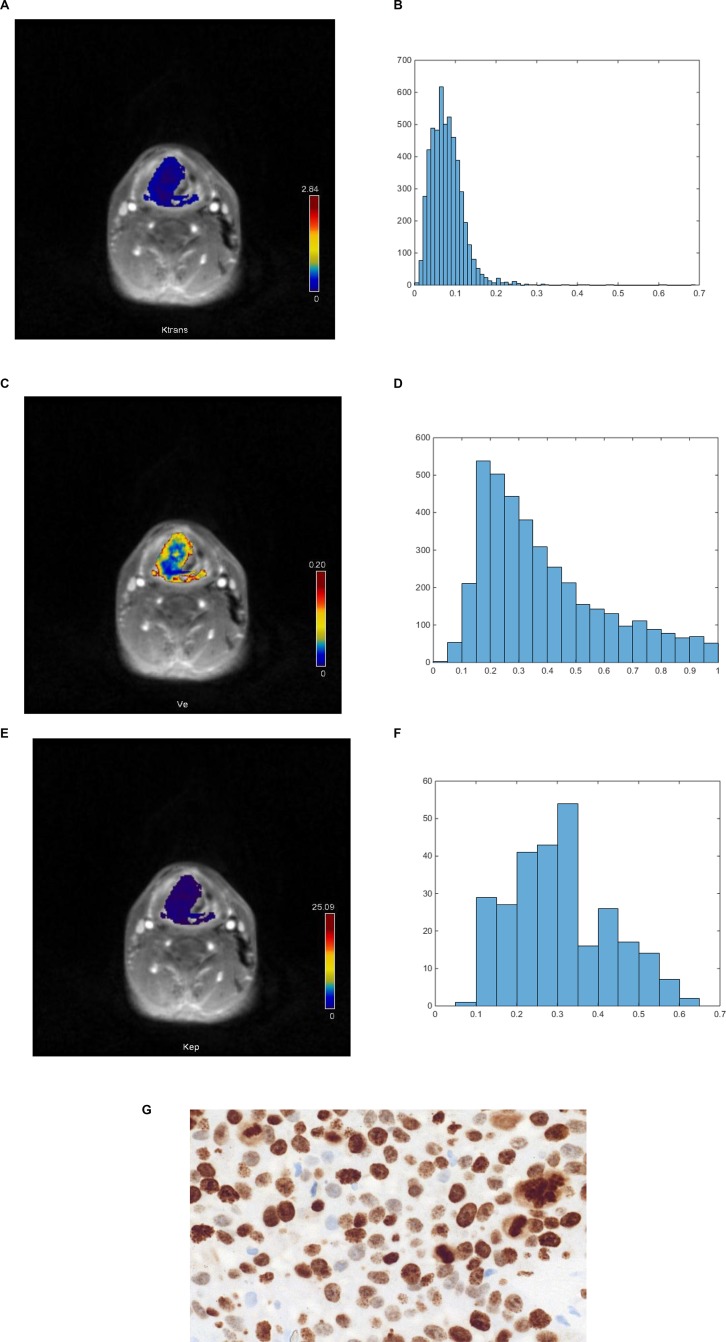
DCE MRI and histopathological findings in a patient with histologically proven squamous cell carcinoma of the oropharynx (**A**) K_trans_ map of the tumor. (**B**) Histogram of K_trans_ values. The histogram analysis parameters (min^-1^) are as follows: mean = 0.078, min = 0.014, max = 0.672, P10 = 0.039, P25 = 0.05, P75 = 0.094, P90 = 0.129, median = 0.07, mode = 0.048, kurtosis = 4.48, skewness = 3.85, entropy = 2.71. (**C**). V_e_ map of the tumor. (**D**) Histogram of V_e_ values. Estimated histogram analysis parameters are as follows: mean = 0.675, min = 0.343, max = 0.992, P10 = 0.466, P25 = 0.532, P75 = 0.822, P90 = 0.929, median = 0.657, mode = 0.475, kurtosis = 1.96, skewness = 0.248, entropy = 2.63. (**E**) K_ep_ map of the tumor. (**F**) Histogram of K_ep_ values. Estimated histogram analysis parameters (min^-1^) are as follows: mean = 0.399, min = 0.14, max = 0.72, P10 = 0.23, P25 = 0.3, P75 = 0.49, P90 = 0.54, median = 0.41, mode = 0.47, kurtosis = 2.47, skewness = 0.059, entropy = 3.31. (**G**). Immunohistochemical stain (MIB-1 monoclonal antibody). Ki 67 index = 90%, cell count = 150, total nucleic area = 46424 μm^2^, average nucleic area = 310 μm^2^.

In all patients dynamic contrast-enhanced (DCE) imaging of the neck was performed. In every case, dynamic T1w DCE sequences (TR/TE 2.47/0.97 ms, slice thickness 5 mm, flip angle 8° C, voxel size 1.2 × 1.0 × 5.0 mm) included 40 subsequent scans à 6 seconds were applicated. Intravenous administration of contrast medium (Gadovist^®^, Bayer Healthcare, Leverkusen, Germany) in a dose of 0.1 mmol per kg of body weight was started after the fifth scan at a rate of 3 ml per second and flushing with 10 ml of normal saline using a power injector (Spectris Solaris, Medrad, Bayer Healthcare, Leverkusen, Germany). Thereafter, all acquired images were transferred to a software module for tissue perfusion estimation (Tissue 4D, Siemens Medical Systems, Erlangen, Germany) as described previously [9, 23, 24]. Though, images of the following pharmacokinetic parameters were saved in DI COM format (Figure 1):

- K_trans_ or volume transfer constant representing the diffusion of contrast medium from the plasma through the vessel wall into the interstitial space;
- V_e_ or volume of the extravascular extracellular space (EES);
- K_ep_ or parameter for diffusion of contrast medium from the EES back to the plasma.

On the next step, the saved DI COM images were processed offline with custom-made Matlab-based application (The Mathworks, Natick, MA, USA). Thereafter, polygonal ROIs were automatically drawn on all of the transferred maps along the contours of the primary tumor on each slice (whole lesion measure) according to the previous description [12]. For every perfusion parameter (K_trans_, V_e_, and K_ep_), mean, maximal, minimal, and median values, as well percentiles 10th, 25th, 75th, and 90th were estimated (Figure 1) according to our previous description (). Furthermore, histogram-based characteristics: kurtosis, skewness, and entropy were also calculated.

### Histopathological analysis

In every case, sections from formalin-fixed and paraffin-embedded tissue were cut at 5 μm and stained with Ki 67 antigen (MIB-1 monoclonal antibody, DakoCytomation, Denmark). Thereafter, the histopathological images were digitalized by using the Pannoramic microscope scanner (Pannoramic SCAN, 3DHISTECH Ltd., Budapest, Hungary) with Carl Zeiss objectives. In every case, the whole sample was acquired at high resolution. Via the integrated Pannoramic Viewer 1.15.4 (open source software, 3D HISTECH Ltd., Budapest, Hungary) the acquired slides were evaluated and three captures with a magnification of ×200 were extracted and saved as uncompressed Tagged Image File Format (TIFF). Furthermore, the digitalized images were analyzed by using ImageJ software 1.48v (National Institutes of Health Image program) with a Windows operating system [27–30]. For this study, the following histopathological parameters were calculated:

- Tumor proliferation index as relation of KI 67 stained nuclei divided by all nuclei [25, 26]. Though for the analysis the area with the highest number of positive tumor nuclei was selected (Figure 1);
- Cell count as a number of all nuclei;
- Total nucleic area (μm^2^) as area of all nuclei;
- Average nucleic area (μm^2^) as a total nucleic area divided by number of nuclei.

### Statistical analysis

Statistical analysis and graphics creation was performed with SPSS 22 (IBM SPSS Statistics for Windows, version 22.0, Armonk, NY: IBM corporation). Values are presented as mean ± standard deviation (SD). The distribution of the acquired data was tested by Shapiro-Wilk test. Mean value comparison was carried out using the Mann-Whitney-*U* test. Spearman's non-parametric rank correlation coefficients were calculated between DCE and different histopathological parameters. Significance level was set at *p* ≤ 0.05.
